# Being a PhD student in Morocco today

**DOI:** 10.7554/eLife.104070

**Published:** 2024-10-10

**Authors:** Anas Bedraoui

**Affiliations:** 1 Faculty of Medical Sciences, Mohammed VI Polytechnic University (UM6P) Benguerir Morocco

**Keywords:** Point of View, Global South, early-career researchers, research culture, scientific publishing

## Abstract

Early-career researchers in the Global South have to overcome obstacles that are not found in high-income countries, but in Morocco at least, the future is looking brighter than the past.

Pursuing a PhD in a low- or middle-income country comes with significant challenges, many of which are not encountered by PhD students in high-income countries. I am a PhD student in Morocco, which is classified as a lower-middle income country by the World Bank. Morocco might be home to the world’s oldest university, Al-Qarawiyyin, but this rich academic history counts for little when confronted with visa restrictions, language barriers, cultural differences and a host of other problems.

## Barriers and obstacles

One of the biggest challenges facing a researcher in Morocco – or anywhere else in the Global South – is obtaining a visa to travel to a high-income country. Last year, for example, I was thrilled when my abstract was accepted for an international conference in Oxford, so I applied for a visa that would allow me to travel to the UK. However, my excitement disappeared when my application was rejected a few weeks later. The decision, made by UK Visas and Immigration on August 4, 2023, was based solely on the documents I had provided (including proof of my PhD enrollment, an invitation letter from the conference, proof of scholarship, and insurance). I was not even invited for an interview, which seemed unfair, especially as I had paid the requisite fee. Moreover, I still have no idea why my application was rejected.

The whole process became a huge source of stress, taking my focus away from my research, and the rejection left me feeling disheartened and demotivated. The frustration from that experience still lingers, and I cannot help but feel anxious about future visa applications. Countless other researchers in the Global South have had similar experiences (Owusu-Gyamfi, 2024; Chugh, 2023).

Job security is a major concern for early-career researchers globally, but in Morocco, additional cultural pressures heighten the challenge. Young men are expected to achieve financial stability quickly, as they are seen as future breadwinners. This financial pressure is tied to traditional views about men having to provide for their family. Meanwhile, women face societal pressure to prioritize marriage and family life, and they are often expected to marry young and fulfill family roles. These different expectations lead to many PhD holders, both men and women, leaving research for more stable, higher-paying jobs – despite their passion for science – as they strive to meet cultural and familial obligations.

The language barrier is also a significant hurdle for many Moroccan PhD students. With English often being their third or fourth language (after Moroccan Darija, Amazigh and French), their ability to pursue postdoctoral opportunities abroad is limited (Housseine and Oifaa, 2020).

Compounding this issue is the underdeveloped research culture in some institutions. Some researchers fall victim to predatory journals that charge high fees, offer no peer review, and provide little-to-no international visibility for their research. I am increasingly convinced that when a PhD student nears the end of their thesis and realizes their research has not been cited because it was published in a predatory journal, they may become susceptible to other predatory services that sell citations in journals (Ibrahim et al., 2024). These predators often target authors in low- and middle-income countries, where the weak research cultures in some institutions make the researchers in these institutions vulnerable. Personally, I receive numerous predatory emails each week, offering invitations to publish in journals with fake impact factors, or to be the keynote speaker at an expensive conference (but only if I pay), or to take part in citation exchanges.

## Signs of progress

Despite these challenges, I am witnessing a positive shift in Moroccan academia. Those who once went abroad as part of the brain drain are now returning home to take up high ranking positions at Moroccan universities. These researchers care about their country and want to make a difference, and their expertise and global connections are helping less experienced researchers in Morocco to overcome the challenges mentioned above.

Morale is also high among PhD holders. In a short informal survey I conducted September, 32/49 respondents rated their experience of doing a PhD in Morocco as 'good' and 5/49 rated it as 'very good', compared with 7/49 for 'bad' and 2/49 for 'very bad'. Reasons for pursuing or not pursing a PhD in Morocco are listed in Table 1.

**Table 1.** Reasons for pursuing or not pursuing a PhD in Morocco. In a short informal survey, researchers were presented with various reasons for pursing and not pursuing a PhD in Morocco. The top five reasons for both options are listed in order in the table, along with the number of researchers who choose each reason. 49 researchers answered this question, and it was possible select more than one reason for each option. More details are available at the following links: [code]; [data].

Reasons to pursue a PhD		Reasons not to pursue a PhD	
Access to diverse research opportunities	28	Limited research funding	36
Growing academic community	23	Limited post-PhD career options	28
Opportunities for international collaboration	22	Fewer advanced facilities	22
Exposure to unique cultural perspectives	13	Limited access to cutting-edge resources	22
Support for innovative projects	10	Bureaucratic hurdles	20

Investment in scientific research is also increasing. Morocco is home to 39 research institutions (according to the Scimago database), and the Minister of Higher Education, Scientific Research, and Innovation allocated $1.412 billion to support research and education this year (Royaume du Maroc, 2023). Additionally, the World Bank recently approved a $300 million loan to help transform higher education and innovation in Morocco (World Bank, 2023; Rahhou, 2023a). And last year it was announced that my university, Mohammed VI Polytechnic University (UM6P) in Benguerir, was due to receive approximately $1 billion from the OCP Foundation (a charitable organization set up by the Office Chérifien des Phosphates, a state-owned mining company; Rahhou, 2023b). The government has also introduced a $700 per month scholarship for PhD students at public universities (Oukerzaz, 2024), with some institutions, such as mine, offering up to $1,000 per month in excellence scholarships (https://www.um6p.ma/fr/doctorat).

Just a few years ago, PhD students in Morocco had to pay for conferences and publications out of their own pocket, often borrowing money, and they had to secure full-time jobs to support their research. Now, everything is changing. We have access to great scholarships, and we no longer need to work other jobs. Our sole mission is to focus on quality research. And although our current situation is not perfect yet, we are on the right path.
